# Shp2 suppresses the adipogenic differentiation of preadipocyte 3T3-L1 cells at an early stage

**DOI:** 10.1038/cddiscovery.2016.51

**Published:** 2016-07-04

**Authors:** J Tao, L Zheng, M Meng, Y Li, Z Lu

**Affiliations:** 1 School of Pharmaceutical Sciences, State Key Laboratory of Cellular Stress Biology, Xiamen University, Xiamen, Fujian, China

## Abstract

Tyrosine phosphatase protein Shp2 is a potential therapeutic target for obesity. However, the mechanism of Shp2 during adipogenesis is not fully understood. The present study investigated the role of Shp2 in the terminal differentiation of preadipocytes. The results showed that Shp2 suppressed adipocyte differentiation in 3T3-L1 cells; overexpression of Shp2 reduced lipid droplet production in 3T3-L1 cells, whereas Shp2 knockdown increased lipid droplet production in 3T3-L1 cells. Furthermore, inhibition of Shp2 activity also enhanced adipocyte differentiation. Interestingly, Shp2 expression was specifically decreased early during differentiation in response to stimulation with the dexamethasone–methylisobutylxanthine–insulin (DMI) hormone cocktail. During the first 2 days of differentiation, Shp2 overexpression impaired the DMI-induced phosphorylation of signal transducer and activator of transcription 3 (STAT3) in 3T3-L1 cells and blocked the peak expression of CCAAT/enhancer-binding proteins *β* and *δ* during preadipocyte differentiation. In conclusion, Shp2 downregulated the early stages of hormone-induced differentiation of 3T3-L1 cells and inhibited the expression of the first wave of transcription factors by suppressing the DMI-induced STAT3 signaling pathway. These discoveries point to a novel role of Shp2 during adipogenesis and support the hypothesis that Shp2 could be a therapeutic target for the control of obesity.

## Introduction

Obesity is a very serious disease that affects a large proportion of the global population.^1^ It not only causes a series of health problems, including high blood pressure, glucose and lipid metabolic disorders, but also increases the incidence of many diseases, such as diabetes, cardiovascular disease and cancer.^2,3^ Obesity involves a very complex pathological process, which makes the prevention and treatment of obesity too difficult to achieve even now.^4^

Disrupted adipogenesis contributes significantly to obesity.^2,5^ A full understanding of the mechanisms underlying adipogenesis could benefit the treatment of obesity.^5,6^ Adipogenesis occurs in two main stages: an initial commitment step, in which cells are restricted to the adipocyte lineage, and the subsequent differentiation of these preadipocytes, governed by a network of transcription factors (TFs), into the adipocyte phenotype.^5,7^ Therefore, two distinct types of adipocyte cell culture models have been developed. C3H10T1/2 cells are the main multipotent stem cell line that can be committed to the adipocyte lineage. 3T3-L1 or F442A cells are preadipocytes that can differentiate into adipocytes.^8,9^

Commitment to the adipocyte lineage is mediated by multiple signaling molecules, including bone morphogenetic protein 4 (BMP4), insulin (INS)-like growth factor 1 (IGF1), interleukin 17, fibroblast growth factor 1, activin, Wnt and hedgehog.^5,6^ Of these molecules, BMP4 is a crucial regulator that can induce the commitment of C3H10T1/2 cells.^10,11^ The initiation of preadipocyte differentiation requires several hormones.^5,12,13^ INS, IGF1, glucocorticoids, triiodothyronine and cAMP are efficient inducers in adipocyte cell culture models.^5,13^ Preadipocytes cultured *in vitro* undergo a pre-confluence proliferation, reach confluence growth arrest and then start hormone-induced clonal expansion.^5,6^ At that time, the cells synchronously re-enter the cell cycle and start to express the first wave of TFs. CCAAT/enhancer-binding proteins *β* and *δ* (C/EBP*β* and *δ*) are the main TFs.^5,6,14^ These TFs are expressed early, during clonal expansion, but are not immediately activated.^12,15,16^ C/EBP*β* and *δ* are phosphorylated by inducers via cytoplasmic mitogen-activated protein kinase (MAPK), cyclin-dependent kinase 2 and glycogen synthase kinase 3*β*, which increase their ability to bind to the promoter regions.^17^ As C/EBP*β* and *δ* achieve maximal DNA-binding activity, they initiate the expression of the second wave of TFs, including C/EBP*α*, peroxisome proliferator-activated receptor *γ* (PPAR*γ*) and sterol regulatory element-binding protein-1c. These TFs induce the expression of lipid synthesis genes, such as adiponectin and fatty acid synthase.^18^

The tyrosine phosphatase Shp2 is a potential target for the treatment of obesity and has a crucial role in glucose and lipid metabolism, and the development of adipose tissues.^19–21^ However, the regulatory effect of Shp2 on these processes is unclear. Several studies in transgenic mice have found that Shp2 activity is important for balancing food intake and energy expenditure, and that deletion of Shp2 in the forebrain causes obesity and diabetes due to disrupted leptin signaling.^22–24^ In contrast, Shp2 also promotes adipogenesis in some studies.^25^ Deletion of Shp2 in embryonic adipose tissue using aP2-Cre blocks the development of adipose tissue in mice.^25^ Shp2 deficiency in embryonic stem cells (ESCs) also suppresses adipogenesis and lipid accumulation.^25,26^ However, the deletion of Shp2 in adipocytes using adiponectin Cre does not show any effects.^25,27^ These conflicting data may be due to the distinct underlying mechanisms of Shp2 in lipid metabolism, adipose tissue development and adipocyte differentiation.^5,9^ Shp2 is a multifunctional signaling protein that usually has dual regulatory effects from single signals (such as INS, growth hormone (GH), transforming growth factor *α* and leptin).^21,28^ As a result, Shp2 may show diverse effects on obesity depending on the context.^21,28^

These conflicting results have limited the study of Shp2 as an effective therapeutic target. Therefore, the roles of Shp2 in the different bioprocesses related to obesity need to be more completely understood to effectively treat this disease by targeting Shp2. Using the 3T3-L1 cell culture model, we demonstrated that Shp2 inhibits early adipocyte differentiation by suppressing the hormone-activated signaling pathway. Our results expand our understanding of Shp2 regulation on adipogenesis and enhance the potential utility of Shp2 as a therapeutic target.

## Results

### Shp2 inhibited the adipogenic differentiation of 3T3-L1 cells

**Figure 1:**
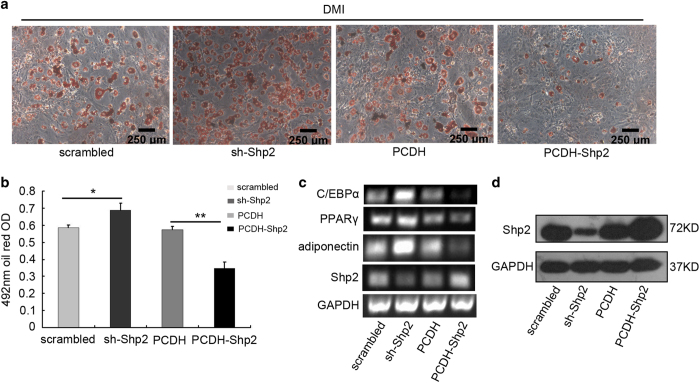
Shp2 mediated the adipogenic differentiation of 3T3-L1 cells. (**a**) After 8 days of inducing adipogenic differentiation, 3T3-L1 cell differentiation was evaluated by the production of lipid droplets, as stained with Oil Red O. The Shp2 protein in 3T3-L1 cells was overexpressed or knocked down using lentivirus, containing PCDH-Shp2 or Shp2 shRNA (sh-Shp2) constructs, respectively. DMI: dexamethasone (Dex), methylisobutylxanthine (IBMX) and insulin (INS). (**b**) Quantification of the lipid content of 3T3-L1 cells at 492 nm. (**c**) The mRNA expression levels of adipocyte differentiation markers in 3T3-L1 cells were evaluated by RT-PCR. (**d**) The protein level of Shp2 in 3T3-L1 cells infected with lentivirus constructs was measured by western blotting. The experiments were replicated at least five times, and one representative result is shown. The quantitative values are the mean±S.E.M. from all replicated experiments. The asterisks denote significance. *P<0.05; **P<0.01.

To determine whether Shp2 regulated the differentiation of preadipocytes, we evaluated the effect of Shp2 protein expression on the differentiation of 3T3-L1 cells. First, 3T3-L1 cells were infected with equivalent titers of the lentivirus overexpression construct PCDH-Shp2 or interfering RNA plasmid sh-Shp2 and their control vectors. Shp2 was successfully overexpressed or knocked down in 3T3-L1 cells (Figure 1d). Cells were then induced to differentiate with the hormone cocktail dexamethasone–methylisobutylxanthine–insulin (DMI), which contains dexamethasone (Dex), methylisobutylxanthine (IBMX) and INS (see details in the Materials and Methods section). On day 8 of treatment, adipocyte differentiation was assessed by staining the lipid droplets with Oil Red O. Abundant lipid droplets accumulated in normal 3T3-L1 cells (Figure 1a, left, first image and right, second image). When Shp2 protein expression was reduced by siRNA, the lipid production was significantly increased (Figure 1a, left, second image). In contrast, the lipid droplets were significantly reduced in cells overexpressing Shp2 protein (Figure 1a, right image). The quantitative assay indicated that the lipid production was increased ~30% in Shp2 knockdown cells, but decreased 50% in Shp2 overexpressing cells (Figure 1b). We also checked the expression of biomarker genes of adipocyte differentiation by PCR with reverse transcription (RT-PCR) and found that knockdown or overexpression of Shp2 up- or downregulated the mRNA levels of C/EBP*α*, PPAR*γ* and adiponectin in differentiated adipocytes, respectively (Figure 1c). These results suggest that Shp2 negatively regulates adipogenic differentiation and that overexpression of Shp2 suppresses the differentiation of preadipocyte 3T3-L1.

### Suppression of Shp2 activity enhanced the differentiation of 3T3-L1 cells

**Figure 2:**
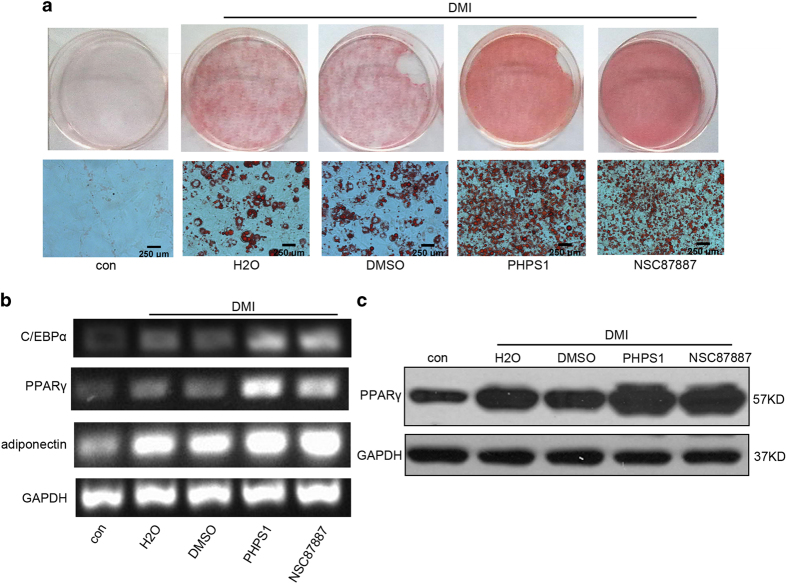
Suppression of the Shp2 activity enhanced the adipogenic differentiation of 3T3-L1 cells. (**a**) Lipid production was evaluated by Oil Red O staining after 8 days of induction of adipogenic differentiation. 3T3-L1 cells were treated with the Shp2 activity inhibitors PHPS1 (10 *μ*M) or NSC87877 (10 *μ*M) during DMI induction for 8 days. (**b**) The mRNA expression levels of adipocyte differentiation markers in 3T3-L1 cells were measured by RT-PCR. (**c**) The protein level of PPAR*γ* in 3T3-L1 cells was measured by western blotting. The experiments were replicated at least five times, and one representative result is shown.

To confirm the regulatory effect of Shp2 on preadipocyte differentiation, we investigated the effects of Shp2 activity inhibitors PHPS1 and NSC87887 on the differentiation of 3T3-L1 cells. PHPS1 or NSC87887 (10 *μ*M) was added to the growth medium during the entire differentiation induction process. At the end of the treatment, adipocyte differentiation was assessed by the production of lipid droplets and the expression of biomarker genes. Oil Red O staining showed that the inhibition of Shp2 activity with PHPS1 and NSC87887 remarkably promoted the production of lipid droplets in adipocytes (Figure 2a). In addition, these inhibitors significantly increased the mRNA levels of biomarker genes PPAR*γ*, C/EBP*α* and adiponectin (Figure 2b). Consistently, PPAR*γ* protein expression was increased by PHPS1 and NSC87887 (Figure 2c). Together, these data demonstrate that the suppression of Shp2 activity also enhances the differentiation of 3T3-L1 cells.

### Shp2 expression was specifically downregulated during the first 2 days of 3T3-L1 differentiation

**Figure 3:**
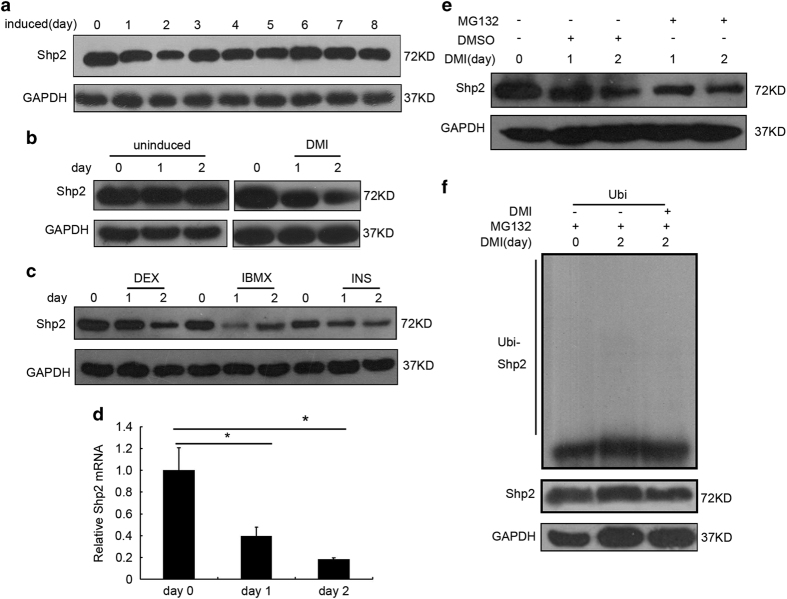
The Shp2 protein was specifically reduced during the first 2 days of the adipogenic differentiation of 3T3-L1 cells. (**a**) The protein level of Shp2 was measured in 3T3-L1 cells by western blotting each day during the normal induction of differentiation. (**b**) Shp2 expression was measured in 3T3-L1 cells treated with each individual inducer (DEX, IBMX or INS). (**c**) Shp2 protein expression was measured in 3T3-L1 cells treated with the three inducers (DMI: DEX, IBMX and INS). Uninduced 3T3-L1 cells served as a control. (**d**) The mRNA level of adipocyte differentiation markers in 3T3-L1 cells was measured by real-time PCR. (**e**) Shp2 expression was measured in 3T3-L1 cells treated with inducers and the proteasome inhibitor MG132. (**f**) The degradation of ubiquitinated Shp2 was detected with a specific antibody in 3T3-L1 cells treated with inducers. The experiments were repeated at least five times, and one representative result is shown. The quantitative values are the mean±S.E.M. from all replicated experiments. The asterisks denote significance. *P<0.05; **P<0.01.

Because Shp2 had a regulatory effect on preadipocyte differentiation, we evaluated the expression pattern of Shp2 during the differentiation of 3T3-L1 cells. The protein level of Shp2 was slightly reduced on the first day (Figure 1a, the second band in the top panel) and reached a significantly lower level on the second day (Figure 1a, the third band in the top panel). However, Shp2 protein recovered to the normal level beginning on the third day (Figure 3a, right six bands in the top panel). As a control, Shp2 expression was not changed in 3T3-L1 cells in the absence of DMI (Figure 3b, left panel). These results indicate that the differentiation inducers DMI reduce the expression of Shp2 protein during the initial stage of preadipocyte differentiation. Interestingly, each inducer also decreased the protein level of Shp2 in cells on the first 2 days of 3T3-L1 differentiation (Figure 3c). In addition, the Shp2 mRNA level was also sharply reduced by the inducers in 3T3-L1 cells during the first 2 days (Figure 3d). Furthermore, the proteasome inhibitor MG132 did not block the reduction of Shp2 protein in cells treated with 10 *μ*M MG132 together with DMI (Figure 3e, right two bands in the top panel). These observations suggest that the differentiation inducers suppressed the gene expression rather than the induced protein degradation of Shp2. As confirmation, Shp2 ubiquitination in 3T3-L1 cells on the second day of differentiation could not be detected with an antibody specific for ubiquitin (Figure 3f).

### Shp2 regulated the early preadipocyte differentiation stage

**Figure 4:**
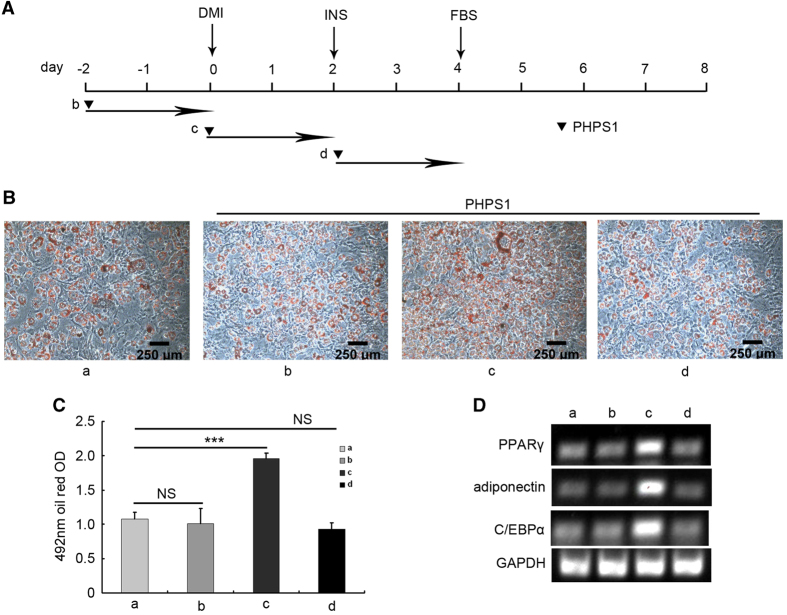
Shp2 regulated the early adipogenic differentiation of 3T3-L1 cells. (**A**) Scheme of the treatment with Shp2 inhibitor PHPS1. (**a**) The normal induction of adipogenic differentiation of 3T3-L1 cells without PHPS1. The cells were treated with 10 *μ*M PHPS1 from day −2 to day 0 (**b**), day 0 to day 2 (**c**) or day 2 to day 4 (**d**). (**B**) The differentiation of 3T3-L1 cells was evaluated based on the production of lipid droplets, as stained with Oil Red O at the end of the induction of differentiation (day 8). (**C**) Quantification of the lipid content in 3T3-L1 cells. (**D**) The mRNA expression levels of adipocyte differentiation markers in 3T3-L1 cells were measured by RT-PCR at day 8. The experiments were replicated at least five times, and one representative result is shown. The quantitative values are the mean±S.E.M. from all replicated experiments. The asterisks denote significance. *P<0.05; **P< 0.01.

Because the expression of *shp2/ptpn11* gene was specifically inhibited during the first 2 days of 3T3-L1 cell differentiation, we hypothesized that Shp2 had a role early in preadipocyte differentiation. To test this hypothesis, the Shp2 inhibitor PHPS1 was added to the induction medium at different time points to assess the effects of Shp2 on each stage of preadipocyte differentiation. The scheme for PHPS1 treatment is illustrated in Figure 4a. As expected, the lipid production was promoted when cells were treated with 10 *μ*M PHPS1 from day 0 to day 2 (Figure 4B and C, c). However, the lipid accumulation was not affected when cells were treated with 10 *μ*M PHPS1 from day −2 to day 0 (Figure 4B and C, b) or day 2 to day 4 (Figure 4B and C, d). Furthermore, the mRNA level of PPAR*γ*, C/EBP*α* and adiponectin was only increased in cells incubated with PHPS1 from day 0 to day 2 (Figure 4D, c). Together, these studies confirm that Shp2 suppresses the preadipocyte differentiation at an early stage.

### Shp2 mediated the cytoplasmic MAPK and STAT3 signaling pathways that are activated by DMI inducers

**Figure 5:**
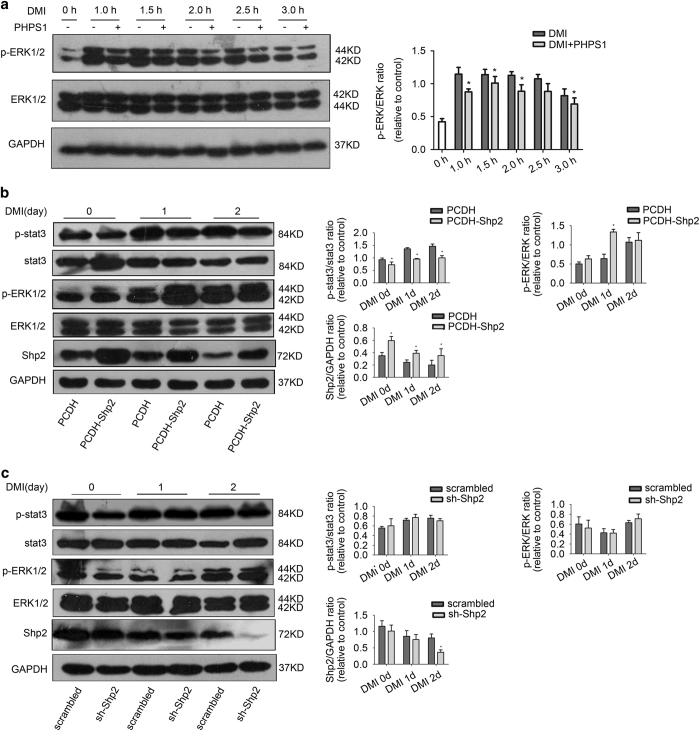
Shp2 inhibited the STAT3 signaling in 3T3-L1 cells early during adipogenic differentiation. (**a**) The level of phosphorylated ERK1/2 induced by DMI with or without Shp2 inhibitor PHPS1 in 3T3-L1 cells. Western blotting was performed with anti-phosphorylated ERK1/2 (Thr202/Tyr204) and ERK1/2 antibodies. (**b** and **c**) The phosphorylation of STAT3 and ERK1/2 were detected by western blotting in 3T3-L1 cells, in which Shp2 was overexpressed or knocked down. Cell lysates were collected from day 0 to day 2. The quantitative values are the mean±S.E.M. from all replicated experiments. The asterisks denote significance. *n*=3; *P<0.05; **P<0.01; *** *P*<0.001.

To identify the underlying mechanisms of Shp2 regulation on preadipocyte differentiation, we evaluated the cytoplasmic signaling pathways activated by DMI in preadipocytes. First, we investigated the effects of Shp2 on DMI-induced MAPK pathway. DMI stimulated the activation of extracellular signal-regulated kinase (ERK) in a time-dependent manner in 3T3-L1 cells; ERK1/2 phosphorylation rapidly increased to a peak level at 1 h (Figure 5a, second band in the top panel) and returned to normal at 3 h of treatment (Figure 5a, right, second band in the top panel). However, the Shp2 inhibitor PHPS1 impaired the stimulatory effects of DMI and reduced DMI-induced ERK1/2 phosphorylation (Figure 5a, third, fifth and seventh bands in the top panel). In contrast, Shp2 overexpression increased the DMI-induced phosphorylation of ERK1/2 on the first day (Figure 5b, fourth band in the third panel). Unexpectedly, DMI-induced activation of ERK1/2 was not affected by the knockdown of Shp2 protein (Figure 5c, the third panel). Next, we examined the regulation of Shp2 on the DMI-induced phosphatidylinositol 3-kinase (PI3K)-protein kinase B (PKB/Akt) pathway, and did not find the effects of Shp2 (data not show).

Furthermore, STAT3 was activated by DMI early during 3T3-L1 cell differentiation (Figure 5b, third and fifth bands in the top first panel). DMI-induced phosphorylation of STAT3 was significantly reduced in preadipocytes that overexpressed Shp2 protein (Figure 5b, fourth and sixth band in the top first panel). However, Shp2 protein knockdown did not further increase STAT3 phosphorylation induced by DMI in 3T3-L1 cells (Figure 5c, top panel).

These results indicate that Shp2 regulates the phosphorylation of ERK and STAT3, but does not affect the activation of Akt induced by DMI during the first 2 days of adipogenic differentiation.

### Shp2 suppressed the peak expression of C/EBP*β* and *δ* early during preadipocyte differentiation

**Figure 6:**
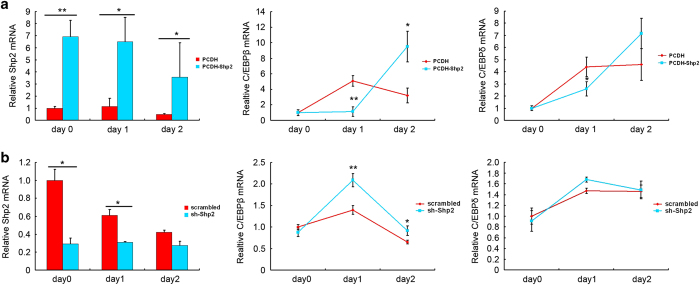
Shp2 suppressed the expression of C/EBP*β* and *δ* early during 3T3-L1 adipocyte differentiation. C/EBP*β*, C/EBP*δ* and Shp2 mRNA expression levels were measured by real-time PCR during the first 2 days of adipocyte differentiation of 3T3-L1 cells that overexpressed (**a**) or underexpressed (**b**) the Shp2 protein. The experiments were repeated at least five times, and one representative result is shown. The quantitative values are the mean±S.E.M. from all replicated experiments. The asterisks denote significance. *n*=3; *P<0.05; **P<0.01; ***P<0.001.

C/EBP*β* and *δ* are part of a first wave of TFs during adipogenesis, and their expression levels peak early during preadipocyte differentiation. We found that Shp2 suppressed the expression of C/EBP*β* and *δ* induced by DMI. The mRNA levels of these TFs were significantly decreased in 3T3-L1 cells that overexpressed Shp2 on day 1 of the differentiation process (Figure 6a, middle and right panels). Unexpectedly, C/EBP*β* and *δ* expression recovered at day 2 (Figure 6a, middle and right panels), which may be due to the reduced expression of Shp2 protein caused by DMI (Figure 6a, left panel). Consistently, C/EBP*β* expression was increased in 3T3-L1 cells with knocked down Shp2 on day 1 (Figure 6b, middle and right panels). These data suggest that Shp2 suppresses C/EBP*β* and *δ* expression and that DMI blocks Shp2’s antagonism to increase C/EBP*β* and *δ* mRNA levels.

## Discussion

Preadipocyte 3T3-L1 is a well-known cell culture model for investigating adipocyte differentiation. In the present study, Shp2 expression was reduced in 3T3-L1 cells during the first 2 days of differentiation. The overexpression of Shp2 decreased the lipid droplets, whereas Shp2 knockdown increased the lipid droplets. These discoveries suggest that Shp2 opposes the early differentiation of adipocytes and supports the hypothesis that Shp2 has a novel role in adipogenesis.

The initiation of preadipocyte differentiation requires hormonal induction.^29^ INS, IBMX (a cAMP phosphodiesterase inhibitor) and Dex (a synthetic glucocorticoid agonist) were combined as an inducer cocktail, and used to treat 3T3-L1 cells for 2 days to induce their re-entry into the clonal expansion and expression of the first wave of TFs (such as C/EBP*β* and *δ*).^5,6,30^ In contrast, Shp2 expression was suppressed by DMI, which indicates that Shp2 has an antagonistic suppressor role during the initiation of preadipocyte differentiation by opposing the hormone inducers. A previous studies also found reduced Shp2 protein expression early in the adipogenic differentiation of 3T3-L1 cells.^31^ Shp2 is a protein tyrosine phosphatase that has inhibitory effects on many factors, including INS and GF.^21^ Thus, INS and other inducers must block the antagonism of Shp2 to trigger signal transduction and the expression of downstream genes during 3T3-L1 differentiation. Interestingly, Shp2 protein expression was restored beginning on the third day of the differentiation process even though cells were still incubated in the presence of INS. This finding indicates that the antagonistic action of Shp2 specifically works early during adipocyte differentiation. As confirmation, an Shp2 inhibitor enhanced the lipid production only in cells treated with PHPS1 during the first 2 days.

A previous studies have shown that Shp2 promotes adipogenesis and that the deletion of Shp2 in adipose tissue before birth in mice blocks the development of adipose tissue.^25^ Similar to the development of other organs, adipose tissue originates from precursor stem cells that are committed to the adipocyte lineage.^5,6^ The underlying mechanism of these processes is different from preadipocyte differentiation.^5,6^ Shp2 is required for the differentiation of ESCs and has crucial roles in the development of several organs.^21,32–35^ Thus, the deletion of Shp2 in adipose tissue in mice would block the production of preadipocytes from adipose stem cells and prevent the development of adipose tissue. Suppression of the Shp2 activity in ESCs also inhibits the adipocyte differentiation by reducing the number of preadipocytes. However, deletion of Shp2 in adipocytes with adiponectin Cre does not affect the development of adipose tissue.^25,27^ These discoveries confirm that Shp2 has a different role during preadipocyte differentiation compared with that during the adipose tissue development.

Shp2 not only suppresses signal transduction via its phosphatase activity, but also triggers cytoplasmic signaling pathways, such as Akt and MAPK.^21,28^ Therefore, Shp2 action involves several cytoplasmic signaling proteins, including Akt, Erk and STAT3.^21,28^ We evaluated the effects of Shp2 on the DMI-induced activation of these signaling proteins and found that Shp2 mediated the phosphorylation of STAT3 and Erk in 3T3-L1 cells, but did not affect the activation of DMI-induced Akt during the first 2 days of adipogenic differentiation. Akt and Erk have important roles in adipogenesis,^36,37^ but their roles in the regulation of the early differentiation of preadipocytes is unclear, especially concerning the expression of the first wave of TFs.

Notably, STAT3 is rapidly activated by adipogenic induction and regulates the transcription of C/EBP*β* early during adipogenesis.^13,38,39^ Both the STAT3-selective inhibitor and JAK2 activity inhibitors suppress the expression of C/EBP*β* and the activation of STAT3 to prevent the adipocyte differentiation.^38^ Furthermore, the present analysis showed that Shp2 significantly inhibited the transcription of C/EBP*β* and *δ* early during 3T3-L1 adipocyte differentiation.^17,18^ C/EBP*β* and *δ* have an important role early in 3T3-L1 adipocyte differentiation.^17,18^ Transcriptional activation of C/EBP*β* initiates the expression of C/EBP*α* and PPAR*γ*, thus initiating the adipocyte differentiation process.^15,40^

**Figure 7:**
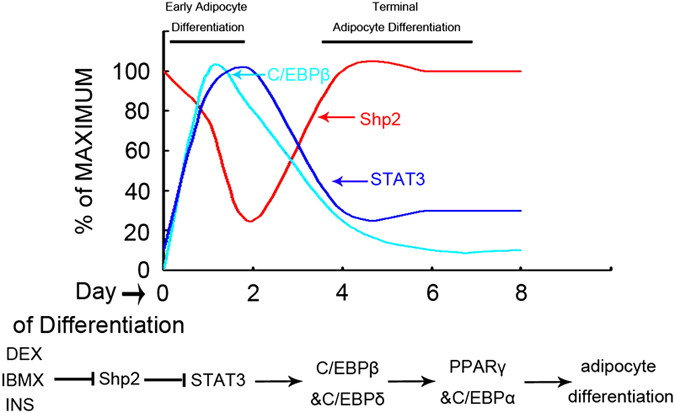
Schematic representation of the negative regulatory effect of Shp2 early during preadipocyte differentiation. The upper panel shows the opposite interaction between Shp2, and the transcription factors C/EBP *β*, C/EBP*δ* and STAT3. The lower panel depicts the signaling pathway mediated by Shp2: the inducers downregulate Shp2 expression, which then suppresses the STAT3 activity to initiate the expression of C/EBP*β* and *δ*, leading to the terminal differentiation of adipocytes.

In summary, Shp2 acts as a suppressor early during the adipogenic differentiation of preadipocytes 3T3-L1. The hormone inducer DMI blocks the inhibitory effect of Shp2 and triggers the peak expression of TFs C/EBP*β* and *δ* to initiate the terminal differentiation of adipocytes (Figure 7). Together, these data demonstrate a novel role of Shp2 in adipocyte differentiation and support the hypothesis that Shp2 could be a therapeutic target for the control of obesity.

## Materials and Methods

### The differentiation model of preadipocytes 3T3-L1

Preadipocyte cell line 3T3-L1 was obtained from the ATCC Company (ATCC-CL-173, Manassas, USA), and cells were cultured in DMEM containing 10% (v/v) heat-inactivated bovine calf serum (1438645, Gibco, Life, Waltham, MA, USA) at 37 °C in a humidified incubator with 5% CO_2_. The growth medium was changed every 2 days. For the differentiation induction, confluent 3T3-L1 cells (defined as day 0) were treated with 0.5 mM IBMX (Sigma, St Louis, MO, USA, I-7018), 1 *μ*M Dex (Sigma, D-4902), 10 *μ*g/ml INS (Sigma, I-5500) and 10% fetal bovine serum (FBS; SV3008702, Hyclone, Thermo Scientific, Waltham, MA, USA) for 2 days. The medium was replaced with DMEM containing 10 *μ*g/ml INS and 10% FBS. After 2 days, cells were cultured in growth medium again for 4 days. For treatment with Shp2 activity inhibitors, cells were incubated with 10 *μ*M PHPS1 (Sigma, P0039) or NSC87877 (Millipore, Billerica, MA, USA, 565851) in growth medium for the indicated time.

### Lentivirus vector construction and infection

The siRNA of Shp2 is targeted on the sequence 5′- GGACATGAATATACCAATATT-3′; the control scrambled sequence is 5′- CCTAAGGTTAAGTCGCCCTCG-3′. The annealed double-stranded fragment (shRNA) was cloned into lentiviral vector pSicoR. To construct the Shp2 overexpression lentivirus, Shp2 mRNA was amplified by PCR from wild-type Shp2 plasmid and inserted into the lentivirus expression plasmid pCDH-copGFP. All constructs were confirmed by DNA sequencing. Plasmids were transfected into 293FT cells to produce lentivirus using a Turbofect transfection reagent. In experiments, cells were infected with equivalent titers of virus. Protein expression was determined by western blotting to ensure the similar expression by the control and experimental viruses.

### Oil Red O staining

3T3-L1 cells were washed three times with PBS and fixed for 1 h with 4% formaldehyde. Cells were washed with 60% isopropanol and incubated with Oil Red O (1320-06-5, Genebase, Shanghai, China) working solution (3 ml/60-mm well) for 10 min at RT. After the Oil Red O solution was removed, cells were immediately washed three times with distilled water. Finally, stained lipid droplets in cells were visualized and photographed. To quantify the lipid production, the Oil Red O-stained lipid droplets were extracted with 100% isopropanol and the OD was measured with an ELISA reader (Bio-Rad, Hercules, CA, USA) at a wavelength of 492 nm.

### RT-PCR and qRT-PCR

Total RNA was extracted from adherent cultured 3T3-L1 cells using TRIzol reagent. cDNA was synthesized using transcript first-strand cDNA synthesis super mix (AT301-02, TransGen Biotech, Beijing, China). For PCR amplification of the cDNA products, the following primer pairs were used: PPAR*γ* forward 5′- GTGCCAGTTTCGATCCGTAGA-3′ and PPAR*γ* reverse 3′- GGCCAGCATCGTGTAGATGA-5′; adiponectin forward 5′- GCACTGGCAAGTTCTACTGCAA-3′ and adiponectin reverse 3′- GTAGGTGAAGAGAACGGCCTTGT-5′; GAPDH forward 5′- TGAA CGGGAAGCTCACTGG-3′ and GAPDH reverse 3′- TCCACCACCCTGTTGCTGTA-5′; and C/EBP*α* forward 5′- GGTGCGTCTAAGATGAGGGA-3′ and C/EBP*α* reverse 3′- CCCCC TACTCGGTAGGAAAA-5′. PCR was performed using a GenePro PCR System (Hangzhou, China). PCR products were electrophoresed by 1% agarose gel electrophoresis and visualized using a Kodak Gel Logic 200 imaging system and the Gene Snap program (Rochester, NY, USA). GAPDH was used as an internal control. The cDNAs were analyzed using the Power SYBR Green PCR kit (04913850001, Roche Diagnostics, Indianapolis, IN, USA) on the ABI StepOne qPCR instrument (Applied Biosystems, Waltham, MA, USA). Each cDNA was amplified (95 °C for 5 s, 58–64 °C for 10 s and 72 °C for 20 s for 40 cycles). All reactions were performed in triplicate, and the data were normalized to GAPDH as an internal control.

### Western blotting and ubiquitination assays

Western blotting was performed as previously described. In brief, cell lysates with equal concentration of total proteins were separated by 10% SDS-polyacrylamide gel electrophoresis, transferred to polyvinylidene difluoride membranes and analyzed by immunoblotting with specific antibodies. Grayscale bands were quantified using Quantity One software (Bio-Rad). For ubiquitination assays, the Shp2-containing complex was first isolated from total cell lysates by immunoprecipitation and separated on SDS polyacrylamide gels. Ubiquitination of Shp2 was detected by western blotting with a specific antibody against ubiquitin (Santa Cruz Biotechnology, sc-9133, Santa Cruz, CA, USA). Antibodies against Shp2 (SH-PTP2, sc-280), PPAR*γ* (sc-7196), *β*-actin (sc-8432) and ERK1 (sc-93) were purchased from Santa Cruz Biotechnology. Antibodies against p-ERK1/2 (4370 s), p-STAT3 (9145 s), STAT3 (9139 s) and GAPDH (2118 s) were obtained from Cell Signaling (Bererly, MA, USA).

### Statistical analysis

All values are shown as the mean±S.E.M. (SPSS 13.0, IBM company, New York City, NY, USA). Paired Student’s *t*-tests were used to compare the mean values, and *P*<0.05 was considered significant.
